# Hepatocellular Carcinoma, Fibrolamellar Variant: Diagnostic Pathologic Criteria and Molecular Pathology Update. A Primer

**DOI:** 10.3390/diagnostics6010003

**Published:** 2015-12-30

**Authors:** Consolato M. Sergi

**Affiliations:** 1 Department of Laboratory Medicine and Pathology, University of Alberta, Edmonton, 8440 112 St., AB T6G2B7, Canada; sergi@ualberta.ca; Tel.: +1-780-407-7951; 2 Department of Pediatrics, Stollery Children’s Hospital, Edmonton, AB T6G2B7, Canada

**Keywords:** liver, tumor, carcinoma, pathogenesis, pathologic diagnosis, criteria

## Abstract

Fibrolamellar hepatocellular carcinoma (FL-HCC) is generally a fairly rare event in routine pathology practice. This variant of hepatocellular carcinoma (HCC) is peculiarly intriguing and,in addition, poorly understood. Young people or children are often the target individuals with this type of cancer. Previously, I highlighted some pathology aspects of FL-HCC, but in this review, the distinctive clinico-pathologic features of FL-HCC and the diagnostic pathologic criteria of FL-HCC are fractionally reviewed and expanded upon. Further, molecular genetics update data with reference to this specific tumor are particularly highlighted as a primer for general pathologists and pediatric histopathologists. FL-HCC may present with metastases, and regional lymph nodes may be sites of metastatic spread. However, peritoneal and pulmonary metastatic foci have also been reported. To the best of our knowledge, FL-HCC was initially considered having an indolent course, but survival outcomes have recently been updated reconsidering the prognosis of this tumor. Patients seem to respond well to surgical resection, but recurrences are common. Thus, alternative therapies, such as chemotherapy and radiation, are ongoing. Overall, it seems that this aspect has not been well-studied for this variant of HCC and should be considered as target for future clinical trials. Remarkably, FL-HCC data seem to point to a liver neoplasm of uncertain origin and unveiled outcome. A functional chimeric transcript incorporating DNAJB1 and PRKACA was recently added to FL-HCC. This sensational result may give remarkable insights into the understanding of this rare disease and potentially provide the basis for its specific diagnostic marker. Detection of DNAJB1-PRKACA seems to be, indeed, a very sensitive and specific finding in supporting the diagnosis of FL-HCC. In a quite diffuse opinion, prognosis of this tumor should be reconsidered following the potentially mandatory application of new molecular biological tools.

## 1. Introduction

Fibrolamellar hepatocellular carcinoma (FLC/FL-HCC) is usually a fairly rare variant of hepatocellular carcinoma (HCC). This variant of hepatocellular carcinoma (HCC) is peculiarly intriguing, and, simultaneously, probably poorly understood. Previously, the FL-HCC was summarized and pathologic features have been illustrated in a personal review [1]. In this review, the distinctive clinico-pathologic features of FL-HCC and the diagnostic pathologic criteria of FL-HCC are fractionally reviewed. Further, molecular genetics update data with reference to this specific tumor are particularly highlighted as a primer for general pathologists and pediatric histopathologists. The target population is not limited to youth only, but childhood and, commonly, adolescents are also affected by this tumor. The epidemiology of this type of cancer shows that both genders are involved and Caucasians are more often the ethnic population affected by this particular type of tumor. In gathering the clinical history of these patients, there is no evidence of parenchymal liver disease. In 2013, Tanaka and colleagues (Tanaka, *et al.* [2]) reported in a memorable article that FL-HCC is a distinct clinical and histologic variant of HCC. Indeed, FL-HCC seems to play a major role in pediatric pathology and hepatology, because it seems to represent almost 1/3 of all pediatric and youth HCCs. FL-HCC usually presents at pediatric age and this has been corroborated by numerous scientific contributions. It is well known that underlying disorders may occur in the setting of HCC [3]. In fact, several databases (PubMed, Scopus, Google) indicate the presence of genetic (hereditary) hemochromatosis, tyrosinemia, endoplasmic reticulum storage disorder of α-1-antitrypsin deficiency as well as progressive familial intrahepatic cholestasis (PFIC) or Byler’s disease as predisposing conditions [1,4,5,6]. A cirrhotic rearrangement of the liver architecture is evident in all above medical conditions, but it is absent in FL-HCC [3,7]. FL-HCC has, conversely, a peculiar *lamellar fibrosis*, *i.e.*, a fibrosis having a substantial thickness, which is considered remarkable for this tumor [1]. This kind of fibrosis occurs without the patients presenting liver cirrhosis or underlying liver disease. It seems to be unique to this kind of cancer. Serum neurotensin, vitamin B_12_ binding capacity (characteristically labelled and known as transcobalamin), and plasmatic γ-carboxy-prothrombin are more often considered laboratory markers for FL-HCC. These markers have been associated with disease burden [8,9,10,11,12]. Serum α fetoprotein (AFP) may be considered during the clinical and laboratory work-up, but it is elevated in only 10% *vs.* 60% of HCC with classic morphology [13,14]. Clinically, individuals harboring FL-HCC have similar symptoms to HCC of classic type, but may also present with two unusual phenotypes, including gynecomastia and Budd-Chiari syndrome [15,16,17,18,19]. In the past, there have been single reports of detection of hepatitis B virus DNA in tumor cells of FL-HCC, but it seems that this event should be considered a coincidental event [20,21,22,23]. To the best of my knowledge, it does not seem that there may be a well determined and specific causal nexus between hepatitis B virus and FL-HCC, but more studies may be necessary once the vaccines against hepatitis B virus infection are diffused worldwide.

Imaging may be fundamental in the diagnostic procedures of hepato-oncology [24,25,26,27,28,29,30]. Interestingly, a central scar may be seen radiologically. This aspect may alert the radiologist in the differential diagnosis to another condition, so-called focal nodular hyperplasia (FNH), which is a benign entity. Radiological experience and databases demonstrate that the FL-HCC scar is often calcified, an important hint, which is uncommonly to be observed with FNH.

## 2. Gross Anatomy and Microscopy

Grossly, FL-HCC is larger than its conventional counterpart (HCC). FL-HCC has an unusual propensity to metastasize and particularly to regional lymph nodes [1,7,31,32,33,34]. FL-HCCs are usually single, hard, scirrhous, and often well-circumscribed. On the cut surface, this kind of tumor is bulging. The color of this tumor is usually white-brown. FL-HCC often shows fibrous bands throughout and a central stellate scar resembling a FNH, as noted radiologically (see above). Although it may occur in both lobes, FL-HCC probably has some uniqueness. FL-HCC is the only liver tumor that is most commonly seen in the left lobe of the liver. It has been variably described as a hemorrhage, necrosis and some bile staining in some cases.

**Figure 1:**
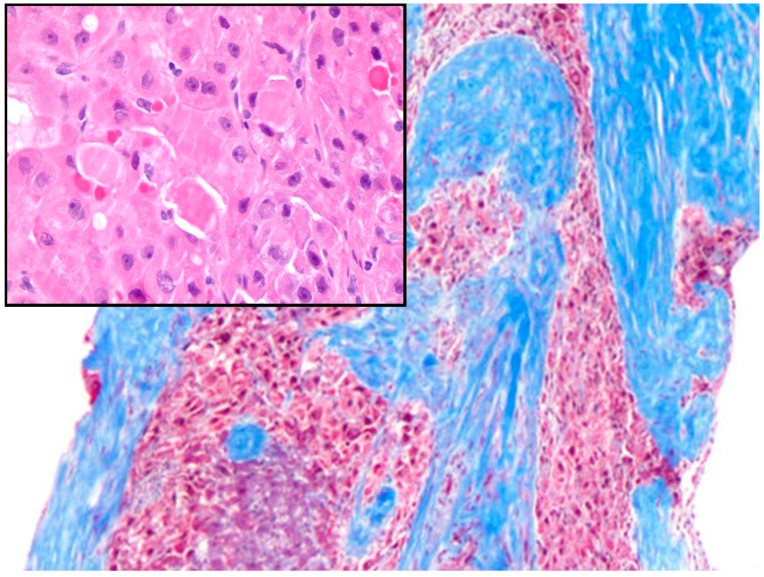
The main picture shows thick fibrous collagen bands, a characteristic finding of the fibrolamellar variant of the hepatocellular carcinoma (Masson’s trichromic, original magnification 50×). The inset shows the high magnification of this kind of tumor with polygonal shape of the cancer cells and the intracytoplasmic pale bodies (Hematoxylin-Eosin staining, original magnification 400×).

The diagnosis of this intriguing tumor is usually performed by light microscopy, which is part of the workup of needle biopsies or surgical open biopsies. FL-HCC shows polygonal cells with large nucleoli and copious eosinophilic cytoplasm. In addition to the scar described above, one of the most striking characteristic is the presence of thick fibrous collagen bands. These bands are, at places, seen to encircle or surround partly the neoplastic hepatocytes (Figure 1). There are additional histological features, which may help in the diagnosis. These include cytoplasmic pale bodies (Figure 1, inset) and depositions of copper as identified by special stains (histochemistry). The routine of pathology practice may be quite challenging and several tools may be in the pathologist’s armamentarium [35]. The sclerotic variant of hepatocellular carcinoma (HCC) is probably its most frequent differential diagnosis in the routine of general pathologists. The peculiar variant of sclerotic HCC may share some “fibrotic” characteristics with FL-HCC. However, the sclerotic variant does not harbor the most characteristic feature of the fibrous collagen bands, which are thick and homogenous. The collagen bands of FL-HCC are often of large thickness in most of the cases encountered in my routine hepatic pathology. In the sclerotic variant, thin collagen fibers are seen instead, and these thin collagen fibers may surround single neoplastic cells. To the best of my knowledge, thick fibrous collagenous bands are usually not seen in the sclerotic variant of HCC. The abundant granular and intensely eosinophilic cytoplasm as demonstrated by light microscopy is revealed to be due to plentiful mitochondria that engulf the cytoplasm. “Ground glass” neoplastic cells often have pale bodies (“ground glass” neoplastic hepatocytes), which show periodic acid Schiff (PAS) positively stained hyaline globules [36]. Other positive markers of FL-HCC include fibrinogen, which seems to have been identified in the “pale bodies”. Bile staining may also be easily observed in FL-HCC using a negative iron stain (Prussian blue stain or Prussian Perl’s stain). In FL-HCC, vascular invasion and necrosis may be seen. Radiologic calcification corresponds exactly to necrosis with foreign body type reaction, which is later recognized under the lens of the general/surgical histopathologist or pediatric pathologist. There are a number of variable features including scattered nuclear pleomorphism and some histologic growth patterns, which may be of trabecular, adenoid or pelioid type. Notably, conventional hepatocellular carcinoma (HCC) may sometimes be found combined with FL-HCC. Regional lymph nodes may be sites of metastatic spread more frequently than conventional HCC. Peritoneum with omentum and lung has also been reported.

**Figure 2:**
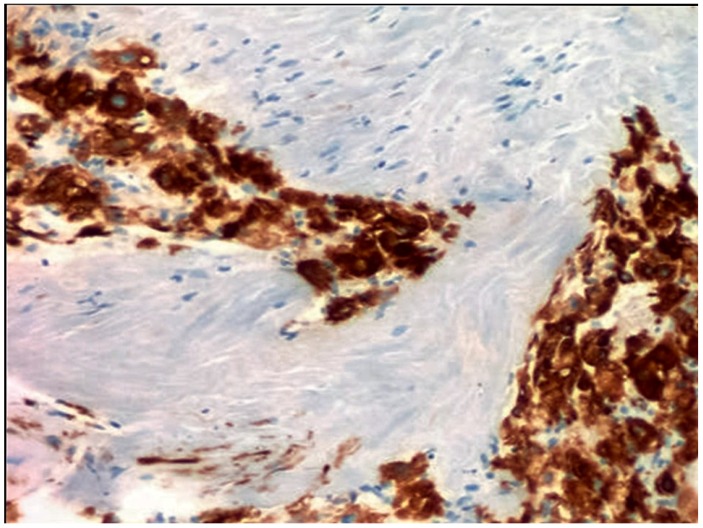
Expression of keratin 7 in fibrolamellar variant of hepatocellular carcinoma (K7 immunohistochemical staining, ABC, original magnification 100×).

**Figure 3:**
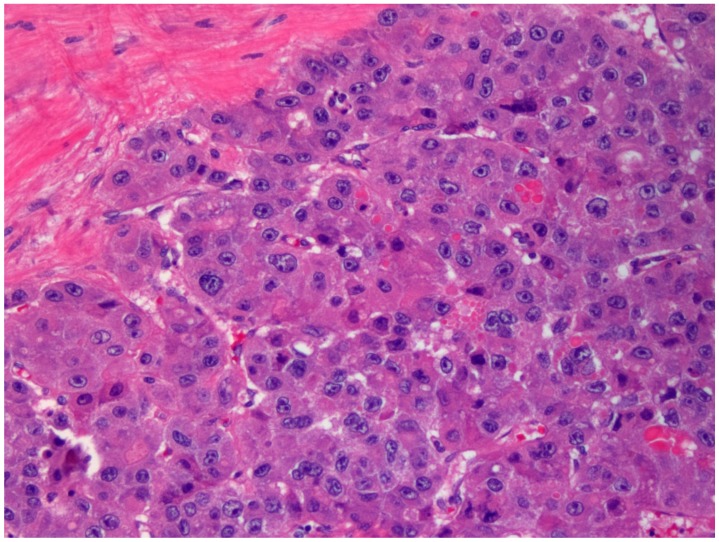
Histology of an intra-omental metastasis of fibrolamellar variant of hepatocellular carcinoma recapitulating the morphology of the original tumor (Hematoxylin-Eosin staining, original magnification 200×).

Immunohistochemically, FL-HCC shows (cyto-) keratin 7 (CK-7 or K-7) (Figure 2), epithelial membrane antigen (EMA), other than Hepatocyte Paraffin 1 (HepPar-1), and CD68. This latter marker or “Cluster of Differentiation 68”, is a glycoprotein which binds to low density lipoprotein and is obviously expressed on monocytes/macrophages [37]. CD68 and carcino-embryonic antigen (polyclonal antibody) or p-CEA are also part of the usually immunohistochemically performed panel [4,34,38,39,40,41,42,43]. In fact, FL-HCC are positive for hepatocellular markers, including HepPar1, p-CEA, and arginase-1, but positivity for glypican-3 varies from 18% to about two-thirds of cases [44,45]. CD68 harbors, indeed, a highly sensitivity, with positivity up to 97% of cases, but specificity may be quite low, because CD68 is seen in 25% of HCC without cirrhosis and in 10% of HCC with cirrhosis [37]. HepPar-1 recognizes a mitochondrial antigen of hepatocytes and has a granular cytoplasmic staining due to a specific binding to mitochondria. Although highly sensitive as a marker identifying the hepatocellular origin of neoplasms, it is negative in higher nuclear grade tumors and this data may be important in investigating the cytology of FL-HCC, which may show a higher degree of atypia. HepPar-1 is moderately specific, although pseudo-positivity may be found in cases with K7 or K20 positivity and chromogranin or synaptophysin positivity. In the majority of cases, HepPar-1 determines the hepatocellular origin of liver neoplasms, particularly using a panel with α-fetoprotein (AFP) and p-CEA or CD10. These markers help differentiate hepatocellular carcinoma from cholangiocarcinoma or metastatic tumors to the liver, as part of a broader panel. An important immunohistochemical finding is the lack of expression of AFP, synaptophysin or chromogranin in FL-HCC. Both synaptophysin and chromogranin are neuroendocrine markers. To the best of my knowledge, the finding of p-CEA in a precise canalicular pattern is specific for HCC. Previously, this phenomenon has been interpreted as probably due to a cross reactivity to biliary glycoprotein I, which is present in bile canaliculi of both normal liver and hepatocellular neoplasms. This has been confirmed in numerous reports and investigations. In my opinion, on this occasion, it is important to remember that monoclonal CEA (m-CEA) is usually negative in FL-HCC as well as in the most often encountered conventional HCC. The peculiar immunophenotype as observed since the beginning of its discovery has suggested the hypothesis that FL-HCC may be a hepatobiliary hybrid neoplasia. Additional markers that may show some positivity in FL-HCC (more constantly that variably, probably and to the best of my knowledge) include CAM5.2 (including K8 and K18), AE1-3, and neurotensin. Considering the literature reviewed until now, it seems that HepPar1, K7, EMA and CD68 are constantly expressed in the bulk of tumors investigated and published and categorized as FL-HCC. Conversely, K19 is usually negative [46]. Growth factor expression seems also quite singular and Ang *et al.* [47] pointed to the expression of epithelial growth factor receptor (EGFR), and Her-2 in FL-HCC. Notably, c-kit and estrogen and progesterone receptor have not been consistently not confirmed by these authors. Haptocorrin, which is also known as transcobalamin-1 (TC-1) or cobalophilin, is a protein that is specifically encoded by the *TCN1* gene in humans. Haptocorrin harbors a vital protective function of the acid-sensitive vitamin B_12_, while it moves through the upper gastrointestinal tract (stomach). Reviewing the literature, haptocorrin has been considered a marker of disease progression [48]. Moreover, focusing their investigation more on new markers, Patonai *et al.* [49] found interesting immunohistochemical findings with regard to claudins. Claudins belong to a family of proteins, which are the most important components of the tight junctions, establishing thus the paracellular barrier that controls the flow of several and different molecules in the highly trafficked intercellular space between the cells of an epithelium. Interestingly, Patonai and colleagues showed that claudins 3, 4, and 7 were not detectable in FL-HCC as in the most part of "conventional" hepatocellular carcinomas (HCC). Conversely, high expression was observed in cholangiocellular carcinomas (CCA). Focal or diffuse expression of claudin 5 was detected in nine out of 11 FL-HCC differently from other tumors or other claudins. In addition, a specific investigation with tricellulin identified that this protein was downregulated in all tumors compared with normal liver [49]. Tricellulin protein is encoded by *MARVELD2* gene. Tricellulin is a membrane protein steadily found at some specific intercellular junctions (tight junctions) between epithelial cells and helps to establish epithelial barriers such as those in the organ of Corti, where these barriers are specifically required for the accomplishment of normal hearing. In fact, from an anatomic point of investigation, the separation of the endo- and perilymphatic spaces of Corti’s organ from one another by epithelial barriers is crucial for normal hearing. Moreover, *MARVELD2* gene defects are at the basis of deafness autosomal recessive type 49 (DFNB49). Although the immunohistochemistry may be considered an expensive tool, at least in some healthcare programs and particularly in countries with low-income, is quite useful in several settings, particularly when settings are different from the usual findings. Conversely, immunohistochemistry is often not used in the case of potential metastatic lesions, because the morphological slide recapitulates slavishly the morphology of the primary tumor (Figure 3).

Cytology may play a major role in the differential diagnosis of hepatic masses [50,51,52,53,54]. Cytologically, FL-HCC is particularly impressive. Most often, the pictorial aspect of FL-HCC has been described to harbor characteristically very large, polygonal cells. These cells have a fairly abundant granular cytoplasm and prominent nucleoli in relatively large nuclei. Some characteristic intracytoplasmic hyaline and pale inclusions as well as intranuclear inclusions have been validated.

Differential diagnosis include a number of diagnoses that may be challenging for the histopathologist and include adenosquamous carcinoma with sclerosis, cholangiocellular carcinoma, which is markedly glandular and mucin positive [55]; focal nodular hyperplasia, which, as indicated above, has smaller size, harbors bile ductular proliferating structures (no-ductal plate malformation alike [56,57,58,59]), which are associated with fibrous stroma and inflammatory cells, but no bile staining grossly and no hepatocytic atypia or pleomorphism obviously; hepatocellular carcinoma, sclerosing or scirrhous variant (SV-HCC), which shows thin fibrotic trabeculae, there is no oncocytic change of the tumor cells, which are smaller in size, and common pseudoglandular pattern is observed (Table 1); metastatic carcinoma with sclerotic stroma (clinical history, electronic medical records); other neuroendocrine tumors (neuroendocrine markers), and paraganglioma, which shows the peculiar Zellballen growth pattern, rounded nuclei with lack of atypia, vascular stroma, but no dense compact fibrosis and, of course, paraganglioma is also positive for neuroendocrine markers [60]. Moreover, a sub-variant of FL-HCC has been remarkably described, which is known as clear cell variant of fibrolamellar carcinoma (FL-HCC, clear cell variant) [61]. In addition to the classic FL-HCC, clear cells are seen in the clear cell variant of FL-HCC. These clear cells are apparently due to ballooning and rarefaction changes of mitochondria, a phenomenon, which have been singularly reported. In particular and more detail, the presence of pseudoacinar structures, mucin expression, and the immunohistochemical finding of K7 positivity may be a dramatic pitfall and FL-HCC can be mistaken for cholangiocellular carcinoma or, even, for metastatic adenocarcinoma. Moreover, the positivity for neuroendocrine markers can lead to the wrong diagnosis of a neuroendocrine carcinoma of the liver, either primary or secondary. However, in our institution and to the best of my knowledge, the expression of hepatocellular markers and the careful adherence to the criteria of the histologic triad help substantially in the differential diagnostic procedure in the majority of cases to promptly address the correct diagnosis.

**Table 1:** Fibrolamellar-hepatocellular carcinoma (FL-HCC) *vs.* Sclerosing Variant of HCC (SV-HCC).

Age	Tumors	
	FL-HCC	SV-HCC
	Youth	Middle-Aged Adults
**Distinguishing Parameters**		
Hx. Chronic Liver Disease/Cirrhosis	(–)	(+)
LN involvement	(±)	(–)
Histologic triad (see notes below)	(+)	(–)
IHC: HepPar1, p-CEA, Arginase-1	(+)	(+)
IHC: CK-7 (K-7) *	(+)	(–)
IHC: NE markers (e.g. NSE, CGA)	(±)	(–)
IHC: Glypican-3	(±)	(+)

Notes: HCC, hepatocellular carcinoma; Hx., history; LN, lymph node (regional); histologic triad: lamellar fibrosis, eosinophilic granular cytoplasm, and prominent nucleolus; IHC, immunohistochemistry; NE, neuroendocrine; NSE, non-specific enolase; CGA, chromogranin A; +, expression present; ± sometimes expression present; −, no expression; *, usually.

## 3. Molecular Pathways

There are typical pathways that are commonly mutated in conventional HCC. These include β-catenin and p53, which are not differentially regulated in FL-HCC. Other factors including RAS, MAPK, EGFR, and PI3K have been investigated. These markers have been found to be upregulated in a subset of affected patients [39,62]. Being a protein superfamily of small GTPases, the Ras superfamily is related to the Ras protein subfamily and the key human members are KRAS, NRAS, and HRAS [63,64,65,66]. Mitogen-activated protein kinases (MAPK) are particular protein kinases with the property to be specific to three amino acidic residues, including serine, threonine, and tyrosine. These protein kinases belong to a well distinct group, called the CMGC (CDK/MAPK/GSK3/CLK) kinase group. Intriguingly, MAPKs are linked to a diverse array of stimuli, such as proinflammatory cytokines, mitogens, osmotic stress, and heat shock. MAPKs seem regulating cell functions including cell proliferation, gene expression, differentiation, mitosis, cell survival, and programmed cell death (apoptosis) [62,67,68,69]. The epidermal growth factor receptor (EGFR), also referred to in humans as ErbB-1 or HER1, is the cell-surface receptor for members of the epidermal growth factor family of extracellular protein ligands. Mutations affecting EGFR expression or activity have been described in cancer [63]. Phosphatidylinositol-4,5-bisphosphate 3-kinase (also called phosphatidylinositide 3-kinases, phosphatidylinositol-3-kinases, PI 3-kinases, PI(3)Ks, PI-3Ks) represent a family of enzymes, which are involved in specific cellular functions, including individual cell growth, cell proliferation and differentiation, intracellular trafficking and cell motility, as well as cell survival. Some of these functions and their regulating proteins are in turn involved in carcinogenesis. In detail, PI3Ks are a family of related intracellular signal transducer enzymes with probably the fine quality to phosphorylate the hydroxyl group (3′-position) of the inositol ring of phosphatidylinositol [68,70,71].

In 2014 and 2015, the molecular biology of FL-HCC has been target of some very extraordinary and memorable articles. Riehle *et al.* [72] have highlighted some molecular pathways. These authors have delineated that mechanistic target of rapamycin complex 1 (mTORC1) is activated in FL-HCC. This finding has been found to be associated with fibroblast growth factor receptor 1 (FGFR1) overexpression in FL-HCC [72]. Molecular profiling of FL-HCC shows three FLC classes with distinct genomic patterns [73] The three classes include the proliferation class (~1/2 of patients), the inflammation class (~1/4 of patients), and the unannotated class (the remaining 1/4 of patients) based on subsequent functional characterization using geneset enrichment analysis (GSEA) and nearest template prediction, and, additionally, immunohistochemistry (IHC). Remarkably, all three classes show expression of genes that regulate neuroendocrine function, as well as histologic markers of cholangiocytes and hepatocytes. Little variation in copy number is recorded for FL-HCC (~1/10 of cases) with the most frequent one being located at 8q24.3 as gene amplification, and at 19p13 (~1/4 of cases) and 22q13.32 (~1/4 of cases) as gene deletions [74]. In about four out of five FL-HCCs cases, it is, indeed, possible to detect the chimeric DNAJB1-PRKACA fusion transcript [75]. Other mutations in *BRCA2* gene (~1/20 of cases) may also occur. There is an 8-gene signature, which has been identified as important in predicting survival of patients with FL-HCC. This 8-gene signature include: *PEAR1*, *KRTAP*, *KLRD1*, *OSBPL8*, *RPL32*, *SLC26A11*, *RGS11*, and *RAPGEF1* [76]. However, it seems that the DNAJB1-PRKACA fusion protein represents the best target for diagnostic and therapeutic advancements. Immunohistochemically, dual cholangiocyte and hepatocyte differentiation pattern of FL-HCCs has been confirmed by the authors. There is, indeed, the uniform positivity of HepPar1 and K7 markers in FLCs, in contrast of K19 and an enrichment of progenitor cell features (EpCAM) in the proliferation class. A wonderful RNA *in situ* hybridization strategy was successively provided by Graham and colleagues in 2015 [74]. This data enabled the affirmation that detection of DNAJB1-PRKACA chimeric transcript is probably a very sensitive and highly specific finding in support of the diagnosis of FL-HCC [77]. Thus, Honeyman and colleagues pinpointed a chimeric transcript that is expressed in FL-HCC but not in adjacent normal liver. This chimeric gene fusion transcript arises as the result of a deletion of about 400-kilobase localized specifically on human chromosome 19. The RNA of this gene fusion transcript codes for a protein, which contains the amino-terminal domain of DNAJB1, which has been identified as a homolog of the molecular chaperone DNAJ. Further, DNAJ is specifically fused in frame with PRKACA, which has been indicated to be the catalytic domain of protein kinase A. Interestingly, a cell culture assay indicated that this chimeric transcript retains kinase activity and its presence in 100% of the FL-HCCs examined may suggest that this genetic alteration contributes to tumor pathogenesis [75]. This marker will be used in the future to help with the diagnosis of FL-HCC and, particularly, in ambiguous sclerosing/scirrhous variants of HCC occurring without predisposing conditions, young adults or harboring an abnormal immunohistochemical pattern of expression.

## 4. Conclusions

From 1956, the date of the first description of this tumor by Edmondson, until late 2015 [46,78,79,80], there has been little progress in research dealing with FL-HCC [4,7]. The 2014 discovery of DNAJB1-PRKACA gene fusion transcript is sensational and is a very sensitive and specific finding in support of the diagnosis of FL-HCC. FL-HCC expresses biliary, hepatocytic and hepatic-progenitor cell markers, and fewer genomic abnormalities than conventional HCC. It may be interesting to further investigate the epigenomics of this very rare tumor. Mitochondria are extremely well represented in the neoplastic cells of FL-HCC. Recently, our academic research group described the mitochondriome of cholangiocellular carcinoma [81]. In our opinion, molecular biology of the mitochondrial genome may add important observations to the full picture of this intriguing neoplasm.
